# Impact of GST and *TP53* gene polymorphisms on susceptibility to end stage renal disease: A case-control analysis

**DOI:** 10.1371/journal.pone.0359809

**Published:** 2026-10-05

**Authors:** Rashid Mehmood, Pei-Chun Chiang, Madiha Rasool, Muhammad Asif, Abdul Ghafar, Mustansar Khalid, Saba Razaq, Muhammad Khaleeq Alam, Iram Fatima, Iqra Jabeen, Sehrish Ashraf, Furhan Iqbal, Chien-Chin Chen

**Affiliations:** 1 Institute of Zoology, Bahauddin Zakariya University, Multan, Pakistan; 2 Division of Nephrology, Department of Internal Medicine, Ditmanson Medical Foundation Chia-Yi Christian Hospital, Chiayi, Taiwan; 3 Department of Zoology, The Women University Multan, Multan, Pakistan; 4 Department of Nephrology, Allama Iqbal Teaching Hospital, Dera Ghazi Khan, Pakistan; 5 Department of Zoology, Ghazi University, Dera Ghazi Khan, Pakistan; 6 Department of Zoology, Emerson University Multan, Multan, Pakistan; 7 Department of Pathology, Ditmanson Medical Foundation Chia-Yi Christian Hospital, Chiayi, Taiwan; 8 Department of Cosmetic Science, Chia Nan University of Pharmacy and Science, Tainan, Taiwan; 9 Doctoral Program in Translational Medicine, National Chung Hsing University, Taichung, Taiwan; 10 Department of Biotechnology and Bioindustry Sciences, College of Bioscience and Biotechnology, National Cheng Kung University, Tainan, Taiwan; Georgetown University Medical Centre, UNITED STATES OF AMERICA

## Abstract

**Purpose:**

End-stage renal disease (ESRD) is a major global non-communicable condition associated with substantial morbidity and mortality. The current investigation aimed to report the association of *GSTM1*, *GSTT1* genotypes and single nucleotide polymorphisms (SNPs) in *GSTP1* (rs1695) and *TP53* (rs1042522) genes with ESRD.

**Methods:**

Clinically confirmed ESRD patients (n = 305) and healthy controls (n = 305) were enrolled from District Multan in Punjab, Pakistan, during 30-10-2023 till 28-02-2024, following their informed verbal consent. Multiplex and T-ARMS PCR based approaches were used to document the genotype at GSTs and SNPs of targeted genes and their associations with ESRD were analyzed individually as well as in combination.

**Results:**

A significant association of absence of *GSTM1,* presence of *GSTT1,* presence of “G” polymorphic allele at rs1695 in *GSTP1,* and the presence of “C” polymorphic allele at rs1042522 in *TP53* gene was observed with ESRD during the present study. Participants having age ranging between 26–40 years, males and smokers suffered more from ESRD.

**Conclusion:**

Genetic variations in the studied GSTs, along with rs1695 in *GSTP1* and rs1042522 in the *TP53* gene, may influence the progression of ESRD.

## 1. Introduction

End-stage renal disease (ESRD) is among the major non-communicable health disorders that have been reported globally and is associated with high rates of morbidity and mortality [1]. The global burden of chronic kidney disease (CKD) has risen markedly over the past few decades, emerging as a major public health concern. Recent estimates suggested that CKD affects more than 10% of the global population [2]. The growing prevalence of diabetes mellitus, hypertension, obesity and aging population has substantially increased the number of individuals progressing to ESRD [3]. The impact of this disease is particularly severe in low and middle income countries, including Pakistan, where delayed diagnosis, limited access to healthcare facilities, and restricted availability of renal replacement therapies further aggravate patient outcomes. Although significant advances have been made in the management of CKD and ESRD, affected individuals continue to experience reduced quality of life, considerable healthcare expenditures, and an elevated risk of cardiovascular complications and premature death [4].

Disease confirmation is based on a variety of biochemical tests and usually targets the markers for the kidney damage along with glamorous filtration rate (GFR) determination. A CKD is confirmed when the subject has less than 60 mL/min GFR with albumin levels higher than 30 mg/ g of creatinine and having abnormal kidney structure for more than last three months [1]. It would be worth mentioning that ESRD is the final stage of CKD and participants having GFR less than 15 mL/min are considered as ESRD patients [2]. If dialysis or kidney transplant is not offered, the situation may become life-threatening [3]. This abnormal kidney function leads to a variety of drastic consequences including extracellular volume overload, dyslipidemia, anemia along with disturbed mineral and bone [4]. In ESRD patients, fluid retention has significant contributions towards ventricular dysfunction, hypertension, and cardiovascular abnormalities [5]. It is the interaction between genes and the environment that decides the likelihood of a subject to develop ESRD. Individuals with family history of this disease are reported to have three to nine time higher risks to develop ESRD [6]. A USA based investigation has reported that ethnic origin influences the onset and progression of ESRD and African American, Hispanic American and Native American families are known to be at higher risk to develop ESRD [7]. Consequently, a better understanding of the genetic and environmental factors contributing to disease susceptibility and progression is essential for improving prevention, early diagnosis, and therapeutic interventions.

Accumulating evidence suggests that oxidative stress plays a pivotal role in the pathogenesis and progression of ESRD [8]. Excessive generation of reactive oxygen species (ROS) and impaired antioxidant defenses contribute to inflammation, endothelial dysfunction and progressive renal injury [9]. Consequently, genetic variations in antioxidant and oxidative stress-related genes have attracted considerable attention as potential determinants of ESRD susceptibility. Nature has blessed us with a variety of chemicals (enzymes, proteins and non-protein) having anti-oxidant potential and among them are the GSTs (Glutathione S-transferases) that are a group of enzymes that plays role in the detoxification of toxic compounds (both endogenous and exogenous) under the oxidative stress conditions [10]. Humans have seven classes of GSTs and *GSTM* (mu), *GSTT* (theta), and *GSTP* (pi) are most extensively studied as the polymorphisms in these genes are known to result is a variety of pathological conditions [11–13]. *Tp53* is a pro-oxidant that contributes to apoptosis following the release of mitochondrial ROS [14]. p53 proteins regulates the cell replication cycle but under the DNA damage situations, this protein halts the cell cycle until this DNA damage is repaired or upon failure it triggers the cell death preventing the proliferation of the mutated cells [15]. Presence or absence of *GSTT1* and *GSTM1* as well as single nucleotide polymorphisms (SNPs) in GSTs and *TP53* gene has been linked with CKD and ESRD in various ethnic populations but they have not been explored among the Pakistani population. Hence, this investigation was designed to find out the association of presence or absence of *GSTM1* and *GSTT1* gene, genotypes at rs1695 in the *GSTP1* and rs1042522 in *TP53* gene with the ESRD in participants that were enrolled from Multan district in Punjab, Pakistan.

## 2. Materials and methods

### 2.1 Participants and sample collection

The study design, experimental protocols, and participant handling procedures were reviewed and approved by the Institutional Ethical Review Board (ERB) of Nishtar Medical University, Multan, Pakistan (Approval No. 18186/NMU). All procedures were conducted in accordance with declaration of Helsinki. Informed verbal consent was obtained from all participants prior to enrollment. The ERB approved the use of verbal consent because a substantial proportion of the study population was elderly and had limited literacy. The consent process was documented in the participant data collection forms. For minor participants, consent was obtained from their parents or legal guardians.

The required sample size was calculated using the formula N = (Z²P(1 − P)D)/e² as described by Jones et al. [16], where Z is the standard normal variate corresponding to a 95% confidence level (1.96), P is the expected prevalence, D is the design effect and e is the desired precision. In the absence of reliable local prevalence estimates, P was set at 0.50 to obtain the maximum sample size. The design effect was assumed to be 1.0 and the margin of error was fixed at 5%. Based on these parameters, the minimum required sample size was 384 participants. A total of 610 individuals were ultimately enrolled, exceeding the calculated requirement and providing adequate statistical power for the genetic association analyses.

Participant recruitment was conducted between 30 October 2023 and 28 February 2024. Clinically confirmed ESRD patients (n = 305) undergoing dialysis at the Nephrology Department of Nishtar Medical University and Hospital, Multan, were enrolled following informed verbal consent. Epidemiological and clinical information was collected using a standardized questionnaire completed at the time of sampling. ESRD diagnosis was established based on clinical evaluation, laboratory investigations, and imaging findings. Diagnostic tests including the Albumin-to-creatinine ratio (ACR: normal range is < 30 mg/g) and blood urea nitrogen (BUN: normal range 7 mg/dL to 20 mg/dL) were performed for each subject followed by a CT scan examination for the diagnosis. The diagnosis of ESRD was confirmed in participants with a glomerular filtration rate (GFR) below 15 mL/min. An equal number of age, sex and ethnicity matched healthy controls (n = 305) without a history of ESRD were recruited from different areas of District Multan following informed verbal consent (S1 Data).

The median cubital vein of each subject was aseptically punctured to collect a blood sample (3–5 ml) that was later used for DNA extraction. From each subject, basic epidemiological and clinical data was collected through a questionnaire, this information included age, gender, marital status, education, family history, hypertension, diabetes, Hepatitis C, smoking, alcohol or drug consumption by the participants.

### 2.2 DNA extraction

Genomic DNA was extracted from the whole blood of each subject by using the inorganic DNA extraction protocol as previously reported [13].

### 2.3 Amplification and genotyping of *GSTT1* and *GSTM1*

In order to report the *GSTT1* and *GSTM1* genotypes among the enrolled cases and controls, a multiplex PCR approach was used as previously reported by Jamil et al. [16]. As there is the concept of presence or absence of these genes, we used the exon 7 of *CYP1A1* gene as an internal control. A total reaction volume of 50 μl was assembled containing 50 ng of template DNA, 5 µl of 10 × PCR buffer, 1.75 mM MgCl_2_, 0.125 mM of each dNTP, and 0.6 mM of each primer (presented in S1 Table) supplemented with 2 µl of Taq DNA polymerase (AbClonal, USA). The amplification protocol for *GSTM1, GSTT1* and *CYP1A1* included an initial denaturation at 94 °C for 5 min, followed by 30 cycles comprising denaturation at 94 °C for 1 min, annealing at 59 °C for 1 min and extension at 72 °C for 1 min, with a final extension step of 10 min at 72 °C [16].

### 2.4 T-ARMS PCR-based amplification of rs1695 in *GSTP1*

Genotypes at rs1695 were explored by using a tetra primer amplification refractory mutation system PCR (T-ARMS–PCR) following Jamil et al. [16]. A 25 µl PCR mixture was prepared containing 50 ng of template DNA, 5 µl of 10 × PCR buffer, 2 mM MgCl_2_, 0.125 mM of each dNTP, and 0.6 mM of each primer(presented in S1 Table) along with 2 µl of Taq DNA polymerase (AbClonal, USA). The final volume was adjusted with double-distilled water. Thermal cycling was performed with an initial denaturation at 95 °C for 5 min, followed by 25 cycles of denaturation at 94 °C for 30 s, annealing at 60 °C for 45 s, and extension at 72 °C for 30 s. A final extension was conducted at 72 °C for 5 min, after which the PCR products were maintained at 4 °C until gel electrophoresis.

### 2.5 T-ARMS PCR-based amplification of rs1042522 in *TP53*

Genotypes at the rs1042522 in the *TP53* gene were determined by using the T-ARMS–PCR technique following Asadi et al. [17]. The PCR assay was conducted in a 25 µl reaction mixture composed of 13 mM Tris–HCl (pH 8.3), 65 mM KCl, 1.5 mM MgCl_2_, 300 µM of each dNTP, 5 units of DNA polymerase (AbClonal, USA), 0.5 µM of each primer (presented in S1 Table) and 5 µl of template DNA. Amplification was carried out under the following program: initial denaturation at 95 °C for 10 min, followed by 35 cycles of 95 °C for 45 s, 56 °C for 30 s, and 72 °C for 45 s, with a final extension step at 72 °C for 10 min [17].

To assess reproducibility, approximately 10% of samples were randomly selected and re-genotyped in independent PCR runs, showing 100% concordance. Negative (reaction mixtures without template DNA) controls were included in each PCR run to ensure assay specificity and exclude contamination.

### 2.6 Agarose gel electrophoresis and documentation

All PCR amplicons were separated on a 1.8% agarose gel. A 100 bp DNA ladder (New England BioLabs, USA) was included to estimate fragment sizes. Electrophoresis was carried out at 120 V and 90 mA for 30 minutes. DNA bands were visualized under UV light using a UV illuminator (Biostep, Germany), and images were captured with a digital camera.

### 2.7 Statistical analysis

Mini Tab (version 19, USA) was used for data analysis. The significance level is set at P ≤ 0.05. Initially, univariate analysis was performed using the Chi-square test to compare genotype and allelic frequencies between cases and controls. To assess the combined effect of *GSTM1, GSTT1*, *GSTP1* (rs1695) and *TP53* (rs1042522) genotypes on ESRD susceptibility, haplotypes were constructed by considering all possible genotype combinations across these loci. The distribution of combined genotypes between cases and controls was evaluated using the Chi-square test. Associations between individual risk factors and disease status were also assessed through Chi square test. This was followed by binary logistic regression model to identify the independent risk factors. While nominal logistic regression was used to present the association between genotypic combinations at rs1695 in *GSTP1* and at rs1042522 in *TP53* with the incidence of ESRD among enrolled cases. Odds ratio (OR) were calculated with 95% confidence intervals (CIs) where required. Hardy Weinberg Equilibrium was calculated for rs1695 in *GSTP1* and rs1042522 in *TP53* to estimate the genetic diversity among the studied population.

## 3. Results

### 3.1 Presence or absence of *GSTM1*and *GSTT1* and their association with ESRD

Multiplex PCR amplified 215 and 480 base pairs fragments upon the presence of *GSTM1* and *GSTT1* genes respectively. Among the *GSTM1* and/or *GSTT1*gene null participants, these bands were missing. For the *CYP1A1* gene, the positive control, a 312 base pair DNA fragment was amplified for all participants (S1 Fig).

Chi-square test results revealed a significant variation of *GSTM1* genotype when compared between ESRD patients and controls (P < 0.001). It was observed that most of the controls had *GSTM1* gene while the majority of the ESRD patients had *GSTM1* null genotype. The odd ratio is indicating that cases have 2.59 fold higher risks than controls to develop ESRD as more cases were *GSTM1* null than controls (Table 1). Similarly, *GSTT1* genotype also varied significantly when compared between ESRD cases and controls (P = 0.05) but contrary to *GSTM1* genotype, it was observed that a higher number of controls had *GSTT1* null genotype than ESRD patients. This was supported by the OR value of 0.69 indicating that the absence of *GSTT1* in cases was not associated with the development of ESRD (Table 1).

**Table 1:** Distribution of presence or absence of *GSTM1* and *GSTT1* gene among ESTD cases and controls enrolled during present study. P-value represents the outcome of Chi square test calculated for each combination.

Genotype	Controls	Case	Chi-Square Value	P-value
*GSTM1* Present	235	172	29.13	P < 0.001***
*GSTM1* Null	70	133	2.59593 (OR)	
*GSTT1* Present	55	73	3.24	0.05 *
*GSTT1* Null	250	232	0.699178 (OR)	

P < 0.05 = Least significant (*); P < 0.001 = Highly significant (***).

OR = Odd ratio.

### 3.2 Genotypic and allelic frequency at rs1695 in *GSTP1* and their association with ESRD

During the amplification of rs1695 in *GSTP1* gene, the outer primers generated a 467 bp amplicon in all participants. While a 233 bp was generated when a homozygous wild (AA) was present while polymorphic (GG) genotype was presented by or 290 bp band. All three bands (467, 290, and 233 bp) were present in participants having heterozygous genotype (AG) at rs1695 (S1 Fig).

When rs1695 genotypes were compared among the enrolled participants, Chi-square test results revealed a significant (P < 0.001) variation among ESRD cases and controls. Wild (AA) genotype was most common (70%) in controls, followed by heterozygous (AG, 23%) and homozygous polymorphic (GG, 7%) genotypes respectively. Among ESRD patients, the AA genotype at rs1695 was the most frequent (59%), followed by AG (24%) and GG (17%). The wild-type (AA) genotype was more frequent among controls, whereas the polymorphic (GG) genotype predominated in ESRD patients. The odds ratio analysis indicated that individuals carrying the GG genotype had a 4.4-fold higher risk of developing ESRD compared with controls (Table 2).

**Table 2:** Genotype and allelic frequency distribution at single nucleotide polymorphism rs1695 in *GSTP1* and in rs1042522 in *TP53* gene among ESRD cases and controls enrolled during presen. Chi-square test was applied to compare the genotype and allelic frequency between case and controls.

Gene/SNP		Genotypic frequency					Allelic frequency			
	Nucleotide	AA	AG	GG	Chi square value	P-value	A	G	Chi square value	P-value
*GSTP1*/rs1695	Controls	214 (70%)	71 (23%)	20 (7%)	31.428	P < 0.001***	499 (82%)	111 (18%)	35.17	P < 0.001***
	Case	179 (59%)	51 (17%)	75 (24%)	4.48324 (OR)		409 (67%)	201 (33%)	2.78 (OR)	
		**GG**	**GC**	**CC**	**Chi square value**	**P-value**	**G**	**C**	**Chi square value**	**P-value**
*TP53*/ rs1042522	Controls	121 (69%)	108 (21%)	76 (10%)	31.282	P < 0.001***	350 (74%)	260 (26%)	50.469	P < 0.001***
	Case	188 (55%)	51 (24%)	66 (21%)	0.49888 (OR)		427 (65%)	183 (35%)	0.576923 (OR)	

P < 0.001 = Highly significant (***).

OR = Odd ratio.

Data analysis revealed that allelic frequency at rs1695 also varied significantly (P < 0.001) upon comparison between ESRD cases and controls. “A” (wild type) allele was most common (82%) among the controls followed by “G” polymorphic allele (18%) (Table 2). A similar trend was observed among ESRD patients [A allele (67%) > G allele (33%)] (Table 2) but the ESRD patients had a significantly higher proportion of polymorphic allele than controls indicating an association of polymorphic allele with the risk to develop ESRD among the enrolled participants during this investigation (Table 2).

### 3.3 Genotypic and allelic frequency at rs1042522 in *TP53* and their association with ESRD

When the rs1042522 in *TP53* was amplified, a 493 bp amplicon was generated by the outer primers among all participants. While 200 bp and 247 bp fragments from *TP53* gene were amplified when homozygous wild (GG) or polymorphic (CC) genotypes were present respectively. Heterozygous genotype (GC) containing participants amplified both 200 and 247 bp products along with 493 bp product generated by the outer primers (S1 Fig).

Chi-square test analysis revealed that the genotypic frequency at rs1042522 significantly (P < 0.001) varied upon comparison between ESRD cases and controls. The homozygous wild type (GG) genotype was more common (69%) among controls followed by heterozygous (CG, 21%) and homozygous polymorphic (CC, 10%) genotypes respectively. Among ESRD cases, the most frequent genotype at rs1042522 was GG (55%), followed by CG (24%) and CC (21%). Our results revealed that polymorphic (CC) and heterozygous (CG) genotypes were more common in cases while wild (GG) genotypes were more common among controls. The OR values indicate that the cases and controls are equally susceptible to develop ESRD (Table 2).

Chi-square test results revealed that allelic frequency at rs1042522 also varied significantly (P < 0.001) among enrolled participants. “G” (wild type) allele was frequent among controls (74%) followed by “C” polymorphic allele (26%) (Table 2). ESRD patients had also similar allelic frequency [G allele (65%) > C allele (35%)] (Table 2) but the ESRD patients had a higher proportion of polymorphic allele than controls indicating that this allele is potentially associated with the risk of developing ESRD based on this investigation (Table 2).

### 3.4 Hardy–Weinberg equilibrium analysis at rs1695 in *GSTP1* and rs1042522 in *TP53*

We calculated heterozygosity observed (HO) and heterozygosity expected (HE) and p value for Hardy–Weinberg equilibrium at rs1695 in *GSTP1* and rs1042522 in *TP53* for both case and control groups. Analysis of our results indicated significant deviations from Hardy–Weinberg Law for both control (x2 = 14.504; P < 0.01 at rs1695 in *GSTP1* and x2 = 23.241; P < 0.01 at rs1042522 in *TP53*) and cases (x2 = 117.838; P < 0.01 at rs1695 in *GSTP1* and x2 = 110.486; P < 0.01 at rs1042522 in *TP53*) indicating genetic diversity in both studied groups for the both screened SNPs (S2 Table).

### 3.5 Combine genotype analysis

In the present investigation, we not only genotyped individual SNPs in the targeted genes and examined their associations with ESRD, but we also analyzed the association between combinations of these genotypes at screened SNPs and ESRD. Our results indicated that participants having either *GSTM1* or *GSTT1* (OR = 1.9) or both *GSTM1* and *T1* null (OR = 2.8) (P = 0.004), participants that were *GSTM1* null and having either AA (OR = 3.68) or GG (OR = 4.58) genotype at rs1695 in *GSTP1* (P < 0.001), *GSTT1* null participants with mutant (GG) genotype at rs1695 in *GSTP1* (OR = 3.15) (P < 0.001) and participants having both GSTT1 and GSTM1 absent with either wild (AA, OR = 3.4) or mutant (GG, OR = 4.5) genotype at rs1695 in *GSTP1* (P < 0.001) were more susceptible to develop ESRD (Table 4).

### 3.6 Association of demographic factors with the incidence of ESRD

Chi-square test results indicated that age (P < 0.001), gender (P = 0.03), and smoking habit (P < 0.001) were the risk factors that were found associated with ESRD during this investigation. Our results showed that males have 1.6 times and smokers have 1.5 time higher risk to develop ESRD. It was also observed that participants in the age range of 26–40 and those having smoking habit were prone to develop ESRD (Table 3).

**Table 3:** Association of the studied demographic factors with the incidence of ESRD among enrolled participants during present investigation. P-value indicates the results of Chi square test calculated for each parameter.

Characteristics	Category	Cases (N = 305)	Controls (N = 305)	P-value
Age (Years)	10-25	49	70	P < 0.001***
	26-40	123	72	0.4097 (OR)
	41-55	91	95	0.730769 (OR)
	56-70	36	66	1.28333 (OR)
	Above 71	6	2	0.23333 (OR)
Gender	Male	192	154	P < 0.001***
	Female	113	151	1.666015 (OR)
Smoking	Yes	65	45	0.03*
	No	240	260	1.564815 (OR)

P < 0.05 = Significant (*), P < 0.001 = Highly significant (***).

OR = Odd ratio.

On the other hand when binary logistic regression analysis was used to compare the risk factors among case and controls, our results indicated that gender was significantly associated with the incidence of ESRD. Males exhibited 1.76-fold higher odds of developing ESRD compared with females suggesting male sex as an independent risk factor in the studied population. In contrast, age and smoking habit did not show a statistically significant association with ESRD indicating that variations in age or smoking were not independently related to ESRD risk in this model (S3 Table).

During present investigation, we also tried to correlate the genotypes of the cases at a specific SNP with the studied risk factors. When the genotype at rs1695 in *GSTP1* gene was correlated with the studied demographic factors, Chi square test results showed that neither gender (P = 0.9), smoking (P = 0.7) or age of cases (P = 0.4) was associated with a particular genotype at this SNP in *GSTP1* gene (S3 Table). Similarly, the genotypes at rs1042522 in *TP53* gene were not associated with the analyzed risk factors among ESRD patients: gender (P = 0.9), smoking (P = 0.3) or the age (P = 0.4) (S4 Table).

Nominal logistic regression showed no significant association between the genotypes at rs1695 in *GSTP1* and demographic or clinical factors in ESRD patients (S5 Table). While no significant association between the genotypes at rs1042522 in *TP53* and demographic or clinical factors in ESRD patients (S6 Table).

## 4. Discussion

Susceptibility to develop a disease varies from person to person and SNPs in our genes are known to affect the severity as well as disease progression [18]. In this investigation, we hypothesized that genotypes and SNPs in GSTs and *TP53* gene are associated with ESRD development among the Pakistani population.

*GSTM1* is a GST gene family member that is involved in the detoxification of environmental carcinogens including those that are present in the tobacco smoke [12]. While *GSTT1* is known to neutralize a wide variety of xenobiotics and endogenous substances and the protein resulting from this gene is playing its role in protecting cells from oxidative stress [11]. Deletions of *GSTM1* and/or *GSTT1* genes result in a deficient antioxidant system, high accumulation of ROS and ultimate loss of kidney function [19]. During the present study absence of *GSTM1* and the presence of the *GSTT1* genotype was found associated with the incidence of ESRD (Table 1). Nomani et al. [20] also reported that the *GSTM1* null genotype was highly associated with ESRD in the Iranian population as the participants lacking this gene had high levels of Malondialdehyde (an indicator of oxidative stress), abnormal lipid metabolism and impaired antioxidant system that resulted in the progression of ESRD. Our results are also in line with Agrawal et al. [21] and Suvakov et al. [22] as they also found *GSTM1* null genotype associated with ESRD in participants enrolled from India and Serbia. Regarding *GSTT1* genotype, contrary to our findings, Yang et al. [23], EL-Said et al. [24] and Suvakov et al. [25] had reported an association of *GSTT1* null genotype with ESRD in participants enrolled from Taiwan, Egypt, and Serbia, respectively. Such a discrepancy may be attributed to population-specific genetic backgrounds, gene–gene or gene–environment interactions, or differences in environmental exposures and study design. These results underscore the importance of considering population heterogeneity in genetic association studies and highlight the need for independent replication to confirm the observed protective effect.

*GSTP1* is involved in the metabolism that results in the detoxification and elimination of foreign complexes that can damage DNA in a cell [13]. In this study, the screened SNP in *GSTP1* gene was found associated with ESRD and polymorphic (GG) genotype was higher among ESRD cases (Table 2). This SNP involves an A-to-G substitution in exon 5 of the *GSTP1* gene, leading to the replacement of isoleucine (Ile) with valine (Val) at the 105th amino acid position of the GSTP1 protein. It has been reported that participants having Valine at codon 105 may have GSTP1 protein that is 2–3 times less stable with reduced detoxification capacity than the protein of the participants having Iisoleucine at this codon resulting in cells exposed to free-radical-mediated toxicity [26]. Similar to our observations, Agarwal et al. (2007) had also reported the association of polymorphic G allele with incidence of ESRD in south east Indian population. Our results are contrary to Nomani et al. [20] and Suvakov et al. [25] as they found that genotypes at rs1695 were not associated with the incidence of ESRD in Iranian and Serbian populations, respectively.

*TP53* is known to plays a critical role repair mechanisms following acute kidney injury (AKI) as the protein encoded by this gene is involve in the regulation of a variety of cellular activities including cell cycle arrest and apoptosis following an insult. P53 protein is known to prevent the progression of AKI to CKD through the activation of PUMA-α and p21 genes [27]. Single nucleotide polymorphism rs1042522 (CGC to CCC transition) is one of the commonly screened SNP in the *TP53* gene that is located in exon 4 and results into an Arginine to Proline amino acid substitution at amino acid position 72 [28]. In the present study rs1042522 in *TP53* gene was found associated with the incidence of ESRD and polymorphic (CC) and heterozygous (CG) genotypes were higher in cases than in controls (Table 2). These are important findings as rs1042522 has never been screened in ESRD patients. Hence, we cannot compare our findings with other ethnic groups but we strongly recommend screening rs1042522 among acute and chronic kidney patients’ across the globe.

It has been established that results generated in studies that deal with SNPs in isolation are not the best disease predictors, as each SNP in a gene alters the function of that particular gene among several genes that normally function together for normal cellular physiology [11]. A single SNP has thus a modest affect in the disease progression and the same biological event is more greatly affected by SNPs in different genes and the disease progression also depends upon how these SNP combinations interact [12]. This haplotype approach allowed assessment of potential additive or interactive effects of multiple loci on disease risk [11]. During current investigation, we found that certain GSTs genotypic combinations significantly increased the risk to develop ESRD among enrolled participants. All the combinations where a particular genotype had significantly higher frequency among the controls were considered protective, while those genotypic combinations having higher frequency among cases were considered as a risk factor of developing ESRD. The results of combination analysis are presented in Table 4. Our results are in agreement with a study from India where Agarwal et al. (2007) had found that participants having null alleles of *GSTM1* and *GSTT1* with mutant allele G alleles at rs1695 in *GSTP1* gene had a higher incidence of ESRD. In a similar case-control study conducted by Suvakov et al. [25], observed highest oxidative stress-mediated damage in participants having *GSTM1*-null/*GSTT1*-null genotype. Patients having four null or low activity genotypes (*GSTM1, GSTT1, GSTP1* and *GSTA1*) had the highest levels of carbonyl and oxidative stress. In a GSTs-based meta-analysis, Omrani et al. [29] had also demonstrated that *GSTM1* null and *GSTM1 and GSTT1* double null genotypes were significantly associated with an increased risk of ESRD, whereas participants having *GSTM1* and/or *GSTT1* were having reduced risk of ESRD. Similarly, when the combinations at rs1695 in *GSTP1* and rs1042522 in *TP53* genes were analyzed, it was observed that participants having wild genotype (GG) at rs1042522 in *TP53* and heterozygous (AG, OR = 6.8) genotype at rs1695 in *GSTP1* gene had higher incidence of ESRD (P < 0.001) (Table 5).

**Table 4:** Genotype and allelic frequency distribution among cases and controls for combinations of GSTs and their possible association with ESRD. Chi-square test was applied to compare the genotype and allelic frequency between case and controls.

Genotype	Control (N = 305)	Case (N = 305)	Chi-Square	P-value
***GSTM1* and *GSTT1***				
Both present	41	21	11.1	0.004**
Either Null	208	203	1.905449 (OR)	
Both Null	56	81	2.82398 (OR)	
***GSTM1* and *GSTP1*(rs1695)**				
M1(+/+) and P1(AA)	179	104	74.9	P < 0.001***
M1(+/+) and P1(AG)	48	25	0.8964 (OR)	
M1(+/+) and P1(GG)	8	43	9.2512 (OR)	
M1(-/-) and P1(AA)	35	75	3.68 (OR)	
M1(-/-) and P1(AG)	23	26	1.9456 (OR)	
M1(-/-) and P1(GG)	12	32	4.58 (OR)	
***GSTT1* and *GSTP1*(rs1695)**				
T1(+/+) and P1(AA)	34	41	41.7	P < 0.001***
T1(+/+) and P1(AG)	16	14	0.72561 (OR)	
T1(+/+) and P1(GG)	5	18	2.9853 (OR)	
T1(-/-) and P1(AA)	180	138	0.635772(OR)	
T1(-/-) and P1(AG)	55	37	0.557871 (OR)	
T1(-/-) and P1(GG)	15	57	3.15122 (OR)	
***GSTT1*, *GSTM1* and *GSTP1* (rs1695)**				
T1 and M1(+/+) and P1 (AA)	28	13	84.2	P < 0.001***
T1 and M1(+/+) and P1 (AG)	11	3	0.5874 (OR)	
T1 and M1(+/+) and P1 (GG)	2	5	5.3846 (OR)	
T1(+/+), M1(-/-) and P1 (AA)	6	28	10.0512 (OR)	
T1(+/+), M1(-/-) and P1 (AG)	5	11	4.7384 (OR)	
T1(+/+), M1(-/-) and P1 (GG)	3	13	9.3333 (OR)	
T1(-/-),M1(+/+) and P1 (AA)	151	91	1.298 (OR)	
T1(-/-), M1(+/+) and P1 (AG)	37	22	1.2806 (OR)	
T1(-/-), M1(+/+) and P1 (GG)	6	38	13.641 (OR)	
T1(-/-), M1(-/-) and P1 (AA)	29	47	3.4907 (OR)	
T1(-/-), M1(-/-) and P1 (AG)	18	15	1.7948 (OR)	
T1(-/-), M1(-/-) and P1 (GG)	9	19	4.547 (OR)	

Abbreviations:

P < 0.01 = Significant (**); P < 0.001 = Highly significant (***).

+/ + represents that two copies specific gene present.

-/- represents that two copies specific gene present.

GSTP1: Glutathione S-transferase pi, GSTM1: Glutathione S-transferase mu, GSTT1: Glutathione S-transferase theta.

OR = Odd ratio.

**Table 5:** Genotype and allele frequency distributions of *GSTP1* rs1695 and *TP53* rs1042522, and their combinations, among cases and controls, to assess their possible association with ESRD. Chi-square test was applied to compare the genotype and allelic frequency between case and controls.

*TP53* rs1042522 and *GSTP1* rs1695	Cases	Controls	Chi-square	P value
*TP53* (GG) and *GSTP1* (AA)	117	105	84.16	P < 0.001 ***
*TP53* (GG) and *GSTP1* (GG)	44	3	0.0759	0.075974 (OR)
*TP53* (GG) and *GSTP1* (AG)	28	13	0.5173	6.809524 (OR)
*TP53* (GC) and *GSTP1* (AA)	33	32	1.0805	2.088578 (OR)
*TP53* (GC) and *GSTP1* (GG)	17	11	0.721	0.667279 (OR)
*TP53* (GC) and *GSTP1* (AG)	16	33	2.2982	3.1875 (OR)
*TP53* (CC) and *GSTP1* (AA)	29	77	2.9586	1.287356 (OR)
*TP53* (CC) and *GSTP1* (GG)	14	6	0.4775	0.16141 (OR)
*TP53* (CC) and *GSTP1* (AG)	17	25	1.6386	3.431373 (OR)

P < 0.001 = Highly significant (***).

OR = Odd ratio.

A number of risk factors including age, gender, race, ethnicity, drug use, smoking, socioeconomic status, hypertension, diabetes mellitus, heart disease, obesity, and increased uric acid have been linked with the incidence of ESRD [30,31]. Our results demonstrated that participants in the age range of 26–40 were more susceptible to develop the ESRD. Our results are partially in line with Süleymanlar et al. [32] as his findings show that participants in rage of 30–39 exhibited a higher risk of ESRD in the Turkey population. Similarly, Wyld et al. [33] had also reported, as a conclusion of their 12-year-long study, that the prevalence of chronic kidney diseases increases with age, with the most rapid growth in people aged 60 years or older. Our results are contrary to Nomani et al. [20] as they had found that age was not associated with the incidence of ESRD in participants enrolled from western Iran. Alkerwi et al. [34] also documented that the prevalence of CKD was significantly associated with increasing age and family history of ESRD in Luxembourg participants. While Kim et al. [35] had also documented that the prevalence of CKD was significantly associated with a family history of ESRD in Korean population.

Analysis of risk factors during the present study indicated that males were more susceptible to ESRD (Table 3). Our results are in line with Harris et al. [36], in their investigation carried out on participants from Australia and New Zealand, had reported that males were more susceptible to ESRD. They found that a higher expression of the epidermal growth factor receptor (EGFR) in males in response to their sex hormones could be a possible reason for their predisposition towards CKD since females had lowered EGFR expression in the kidneys of adult females. Our results are in line with Fukuhara et al. [37] as their results showed that males from Okinawa were more susceptible to ESRD. Our findings are contrary to the Dedinska et al. [38] as they had reported that females were more susceptible to ESRD than males in the Slovakian population. Similarly, Wyld et al. [33] documented that females were more susceptible to ESRD in participants enrolled from Sydney, Australia.

We also observed that participants having smoking habit were more susceptible to ESRD (Table 3). Haroun at al [39]. had reported that smoking habit was associated with the development of CKD in participants enrolled from Washington County in USA. Xia et al. [40] had also identified Cigarette smoking as one of the most significant risk factors for CKD development. Our study also showed that age was also significantly associated with the incidence of ESRD (Table 3). These variations among the studies cited above are due to the different ethnic origin of the enrolled participants as it is well known fact that populations that differ from each other regarding their religion, culture or ethnicity also differ in their illness behaviors due to their differential gene expression [41,42].

In the present study, both the screened SNPs exhibited significant deviations from Hardy-Weinberg equilibrium (HWE) in cases and controls (S2 Table). While deviations from HWE can sometimes indicate genotyping errors, in our study, genotyping was performed using validated protocols with reproducibility checks, making technical error an unlikely explanation. Several biological and population level factors may contribute to such deviations. Our cohort represents a relatively specific ethnic population, and underlying genetic background differences, population stratification, nonrandom mating, or substructure could influence genotype distributions. In addition, evolutionary forces such as natural selection, genetic drift, and gene flow may alter allele frequencies and disturb HWE in small or isolated populations [43–45]. Therefore, we believe that the observed deviations likely reflect true biological variation within this population rather than technical artifacts, highlighting the relevance of these SNPs in relation to the studied disease. Nevertheless, future studies with larger and more diverse cohorts are warranted to validate these associations and to further explore the influence of population-specific factors on HWE deviations.

The small sample size is a limitation of this study as the higher sample size makes the results better predictor and more reliable. We have targeted 4 genes and their SNPs in this investigation, the number can be enhanced in future studies as many other genes and SNPs are reported to be associated with end stage renal disorder in various ethnic groups globally. However, the present study is valuable as these SNPs have never been screened in local population with reference to this growing health concern: end stage renal disorder. The observed associations of *GSTM1, GSTT1, GSTP1* and *TP53* genotypes with ESRD risk may reflect the underlying biological mechanisms related to oxidative stress and detoxification. Absence or reduced activity of specific GST isoforms may increase susceptibility to kidney injury and progression to ESRD. Gene-gene interactions between these loci may further modulate individual risk. While our cohort represents a relatively homogeneous ethnic population, population stratification cannot be completely ruled out and may influence allele frequencies and observed associations. Nevertheless, these findings provide a basis for considering personalized interventions, such as enhanced monitoring, lifestyle modification, or targeted preventive strategies for individuals carrying high risk genotypes. Future studies in diverse populations are warranted to confirm these associations and explore their potential utility in precision medicine approaches for ESRD.

## 5. Conclusion

In conclusion, we are reporting that *GSTM1, GSTT1* genotypes as well as genotypes at rs1695 in *GSTP1* and rs1042522 in *TP53* genes were associated with the incidence of ESRD among the enrolled participants from Pakistan. Males and participants with age between 26–40 years and smokers were more susceptible to develop ESRD. Taking the small subject number enrolled in this study as a limitation, we recommend conducting similar but large-scale studies in various parts of Pakistan to further confirm the association of studied genes and SNPs with the incidence of ESRD in the local population as the investigation performed in larger patient population would be eligible for more meaningful results.

## Supporting information

S1 Table Oligonucleotide primers used for the genotyping of *GSTM1*, *GSTT1*, *GSTP1* and *TP53* genes and for the amplification of *CYP1A1* gene among the enrolled participants. (DOC)

S2 Table Genotype and allele frequencies, Heterozygosity and x^2^ value at single nucleotide polymorphism rs1695 in *GSTP1* and rs1042522 in *TP53* gene among case and controls enrolled during the present study. P-value represents the output of Hardy-Weinberg equation calculated for case and controls.
(DOC)

S3 Table Association of *GSTP1* rs1695 and *TP53* rs1042522 genotypic combinations with risk factors in patients with ESRD. P-value is output of Chi-square test results.
(DOC)

S4 Table Association of genotypic combinations at rs1695 in *GSTP1* and rs1042522 in *TP53* with demographic and clinical factors in ESRD cases. P-value is output of Chi-square test results.
(DOC)

S5 Table Association of genotypic combinations at rs1695 in *GSTP1* gene and the studied demographic factors in patients with ESRD. P-value is output of nominal logistic regression test.
(DOC)

S6 Table Association of genotypic combinations at rs1042522 in *TP53* gene and the studied demographic and clinical factors in patients with ESRD. P-value is output of Nominal Logistic Regression test.
(DOC)

S1 Fig Agarose gel electrophoresis based genotyping results. A. Multiplex PCR based amplification of *GSTT1* and M1 gene. All lanes presenting 312 bp band specific for *CyP1A1* gene (control). Lanes presenting 480 band specific for *GSTT1* gene. Lanes presenting 218 bp band specific for *GSTM1* gene. DNA ladder of 100 bp and in some gels 50 bp was used for size comparison. B. T-ARMS PCR based genotyping at rs1695 in *GSTP1*. Lanes presenting 290 bp band specific for mutant genotype (GG) and lanes presenting 233 bp band specific for wild genotype (AA). While lanes showing both 233 and 290 bp specific for heterozygous (AG) genotype. DNA ladder of 100 bp and in some gels 50 bp was used for size comparison. C. T-ARMS PCR based genotyping at rs1042522 in *TP53*. DNA ladder of 100 bp and in some gels 50 bp was used for size comparison. Lanes presenting 200 bp band specific for wild genotype (GG), lanes presenting 247 bp specific for mutant genotype (CC) while lanes showing both 200 and 247 bp specific for heterozygous (CG) genotype.
(DOC)

S1 Data Individual-level demographic, clinical and laboratory data used for the analyses presented in the manuscript. (XLS)
