# Supplementary material for: Impact of GST and TP53 gene polymorphisms on susceptibility to end stage renal disease: A case-control analysis
Source: PLoS One. 2026 Oct 5;21(10):e0359809. doi: 10.1371/journal.pone.0359809 (PMC13637908; doi:10.1371/journal.pone.0359809)
Supplement: Supplementary file 1 [file pone.0359809.s001.doc]

**Supplementary Table 1.** Oligonucleotide primers used for the genotyping of *GSTM1*, *GSTT1*, *GSTP1* and *TP53* genes and for the amplification of *CYP1A1* gene among the enrolled subjects.

| **Targeted gene and SNP** | **Primer Name** | **Primer sequence (5′-3′)** | **Amplicon** | **Annealing Temperature (ᵒC)** | **Reference** |
| --- | --- | --- | --- | --- | --- |
| *GSTM1* | GSTM1 Reverse | GTTGGGCTCAAATATACGGTGG | 215 bp |  | [16] |
|  | GSTM1 Forward | GAACTCCCTGAAAAGCTAAAGC |  |  |  |
| *GSTT1* | GSTT1 Reverse | TCACGGGATCATGGCCAGCA | 480 bp | 59 |  |
|  | GSTT1 Forward | TTCCTTACTGGTCCTCACATCTC |  |  |  |
| *CYP1A1* | CYP1A1 Reverse | CAGCTGCATTTGGAAGTGCTC | 312 bp |  |  |
|  | CYP1A1 Forward | GAACTGCCACTTCAGCTGTCT |  |  |  |
| *GSTP1* (rs1695) | Outer Reverse | ATAAGGGTGCAGGTTGTGTCTTGTCCCA | Outer primers 467bp |  | [16] |
|  | Outer Forward | CAGGTGTCAGGTGAGCTCTGAGCACC | A allele 233 bp | 62 |  |
|  | Inner Reverse | GCTCACATAGTTGGTGTAGATGAGGGATAC | G allele 290 bp |  |  |
|  | Inner Forward | CGTGGAGGACCTCCGCTGCAAATCCA |  |  |  |
| *TP53* (rs1042522) | Outer Reverse | TGCAGGGGGATACGGCCAGGCATTGAAGTC | Outer primers 493bp |  | [17] |
|  | Outer Forward | TGGGGGGCTGAGGACCTGGTCCTCT | G allele 200 bp | 56 |  |
|  | Inner Reverse | GCTGCTGGTGCAGGGGCCAGGG | C allele 247 bp |  |  |
|  | Inner Forward | CCAGAATGCCAGAGGCTGCTCCGCG |  |  |  |
