# Supplementary material for: Impact of GST and TP53 gene polymorphisms on susceptibility to end stage renal disease: A case-control analysis
Source: PLoS One. 2026 Oct 5;21(10):e0359809. doi: 10.1371/journal.pone.0359809 (PMC13637908; doi:10.1371/journal.pone.0359809)
Supplement: Supplementary file 2 [file pone.0359809.s002.doc]

**Supplementary Table II.** Genotype and allele frequencies, Heterozygosity and x^2^ value at single nucleotide polymorphism rs1695 in *GSTP1* and rs1042522 in *TP53* gene among case and controls enrolled during the present study. P-value represents the output of Hardy-Weinberg equation calculated for case and controls.

| **SNP/Gene** | **Treatment** | **Genotypic frequency** | | | **Allelic frequency** | | x^2^ Value | P-value |
| --- | --- | --- | --- | --- | --- | --- | --- | --- |
|  |  | **AA** | **AG** | **GG** | A | G |  |  |
| rs1695 in *GSTP1* | Controls (HO) | 214 | 71 | 20 | 499 | 111 | 14.504 | P < 0.01 ** |
|  | HE | 204.09 | 90.8 | 10.09 | 0.82 | 0.18 |  |  |
|  | Case (HO) | 179 | 51 | 75 | 409 | 201 | 117.838 | P < 0.01 ** |
|  | HE | 137.12 | 134.77 | 33.12 | 0.67 | 0.32 |  |  |
|  |  | **GG** | **GC** | **CC** | **G** | **C** |  |  |
| rs1042522 in *TP53* | Controls (HO) | 121 | 108 | 76 | 350 | 260 | 23.241 | < 0.01 ** |
|  | HE | 100.41 | 149.18 | 55.41 | 0.57 | 0.43 |  |  |
|  | Case (HO) | 188 | 51 | 66 | 427 | 183 | 110.486 | P < 0.01 ** |
|  | HE | 149.45 | 128.1 | 27.45 | 0.7 | 0.3 |  |  |

HO = Observed heterozygosity, HE = Expected heterozygosity, P < 0.001 = Significant (**)
