# Supplementary material for: Impact of GST and TP53 gene polymorphisms on susceptibility to end stage renal disease: A case-control analysis
Source: PLoS One. 2026 Oct 5;21(10):e0359809. doi: 10.1371/journal.pone.0359809 (PMC13637908; doi:10.1371/journal.pone.0359809)
Supplement: Supplementary file 3 [file pone.0359809.s003.doc]

**Supplementary Table 3.** Association of the studied demographic factors with the incidence of ESRD among enrolled subjects during present investigation. P-value indicates the results of binary logistic regression.

| **Risk factor** | **Category** | **OR (**95% CI)**)** | **P-value** |
| --- | --- | --- | --- |
| Age | Various age groups | 1.0078 (0.9968-1.0189) | 0.167 |
| Gender | Male vs female | 1.76 (1.2757-2.4383) | 0.001 ** |
| Smoking habit | Smoker vs non smoker | 1.01 (0.6405-1.5953) | 0.963 |

P < 0.05 = Significant (*), P < 0.001 = Highly significant (***)

OR = Odd ratio; 95% CI = 95% confidence intervals
