# Supplementary material for: Impact of GST and TP53 gene polymorphisms on susceptibility to end stage renal disease: A case-control analysis
Source: PLoS One. 2026 Oct 5;21(10):e0359809. doi: 10.1371/journal.pone.0359809 (PMC13637908; doi:10.1371/journal.pone.0359809)
Supplement: Supplementary file 4 [file pone.0359809.s004.doc]

**Supplementary Table 4.** Association of genotypic combinations at rs1695 in GSTP1 and rs1042522 in TP53 with demographic and clinical factors in ESRD cases. P-value is output of Chi-square test results.

| **Gene/SNP** | **Risk factors** | **Categories** | **AA genotype** | **GG genotype** | **AG genotype** | **Chi-square** | **P –value** |
| --- | --- | --- | --- | --- | --- | --- | --- |
| *GSTP/* rs1695 | Age | 10-30 | 52 | 14 | 14 | 6.3 | 0.4 |
|  |  | 31-50 | 93 | 40 | 31 |  |  |
|  |  | 51-70  71-100 | 30  4 | 19  2 | 6  0 |  |  |
|  | Gender | Male | 112 | 48 | 32 | 0.04 | 0.9 |
|  |  | Female | 67 | 27 | 19 |  |  |
|  | Smoking | Yes | 24 | 13 | 7 | 0.7 | 0.7 |
|  |  | No | 155 | 62 | 44 |  |  |
| *TP53*/ rs1042522 | Age | 10-30 | 52 | 11 | 15 | 6.3 | 0.4 |
|  |  | 31-50 | 101 | 38 | 26 |  |  |
|  |  | 51-70  71-100 | 33  2 | 14  3 | 9  1 |  |  |
|  | Gender | Male | 120 | 40 | 32 | 0.2 | 0.9 |
|  |  | Female | 68 | 26 | 19 |  |  |
|  | Smoking | Yes | 23 | 13 | 8 | 2.3 | 0.3 |
|  |  | No | 165 | 53 | 43 |  |  |

P ˃ 0.05 = Non-significant
