# Supplementary material for: Impact of GST and TP53 gene polymorphisms on susceptibility to end stage renal disease: A case-control analysis
Source: PLoS One. 2026 Oct 5;21(10):e0359809. doi: 10.1371/journal.pone.0359809 (PMC13637908; doi:10.1371/journal.pone.0359809)
Supplement: Supplementary file 5 [file pone.0359809.s005.doc]

**Supplementary Table 5.** Association of genotypic combinations at rs1695 in GSTP1 gene and the studied demographic factors in patients with ESRD. P-value is output of nominal logistic regression test.

| **Predictor** | **Coef** | | **SE Coef** | | **Z** | | **P** | | **Odds Ratio** | | **95% CI Lower** | | **95% CI Upper** | |
| --- | --- | --- | --- | --- | --- | --- | --- | --- | --- | --- | --- | --- | --- | --- |
| **Comparison AG vs AA** | |  | |  | |  | |  | |  | |  | |  |
| Constant | 1.97941 | | 6.80066 | | 0.29 | | 0.771 | |  | |  | |  | |
| Gender | -0.150056 | | 0.363606 | | -0.41 | | 0.680 | | 0.86 | | 0.42 | | 1.76 | |
| Age | -0.0204536 | | 0.0141937 | | -1.44 | | 0.150 | | 0.98 | | 0.95 | | 1.01 | |
| Smoking | -0.0773930 | | 0.490682 | | -0.16 | | 0.875 | | 0.93 | | 0.35 | | 2.42 | |
| **Comparison GG vs AA** |  | |  | |  | |  | |  | |  | |  | |
| Constant | -9.44879 | | 5.57447 | | -1.70 | | 0.090 | |  | |  | |  | |
| Gender | 0.0802697 | | 0.312070 | | 0.26 | | 0.797 | | 1.08 | | 0.59 | | 2.00 | |
| Age | 0.0222228 | | 0.0108699 | | 2.04 | | 0.041 | | 1.02 | | 1.00 | | 1.04 | |
| Smoking | -0.278410 | | 0.401075 | | -0.69 | | 0.488 | | 0.76 | | 0.34 | | 1.66 | |

**Coef** = Regression coefficient; **SE Coef** = Standard error of the coefficient; **Z** = Test statistic (Coef ÷ SE); **P** = P-value; OR = **Odds Ratio; 95% CI Lower / Upper** = Confidence interval for the OR.
