# Supplementary material for: Impact of GST and TP53 gene polymorphisms on susceptibility to end stage renal disease: A case-control analysis
Source: PLoS One. 2026 Oct 5;21(10):e0359809. doi: 10.1371/journal.pone.0359809 (PMC13637908; doi:10.1371/journal.pone.0359809)
Supplement: Supplementary file 6 [file pone.0359809.s006.doc]

**Supplementary Table 6.** Association of genotypic combinations at rs1042522 in *TP53* gene and the studied demographic and clinical factors in patients with ESRD. P-value is output of Nominal Logistic Regression test.

| **Predictor** | **Coef** | | **SE Coef** | | **Z** | | **P** | | **Odds Ratio** | | **95% CI Lower** | | **95% CI Upper** | |
| --- | --- | --- | --- | --- | --- | --- | --- | --- | --- | --- | --- | --- | --- | --- |
| **Comparison GC vs GG** | |  | |  | |  | |  | |  | |  | |  |
| Constant | -17.7738 | | 7.71688 | | -2.30 | | 0.021 | |  | |  | |  | |
| Gender | -0.239909 | | 0.424003 | | -0.57 | | 0.572 | | 0.79 | | 0.34 | | 1.81 | |
| Age | -0.0093478 | | 0.0147684 | | -0.63 | | 0.527 | | 0.99 | | 0.96 | | 1.02 | |
| Smoking | 0.207324 | | 0.530873 | | 0.39 | | 0.696 | | 1.23 | | 0.43 | | 3.48 | |
| **Comparison GC vs GG** |  | |  | |  | |  | |  | |  | |  | |
| Constant | -8.25709 | | 5.65995 | | -1.46 | | 0.145 | |  | |  | |  | |
| Gender | -0.329423 | | 0.325621 | | -1.01 | | 0.312 | | 0.72 | | 0.38 | | 1.36 | |
| Age | -0.0153063 | | 0.0115019 | | -1.33 | | 0.183 | | 0.98 | | 0.96 | | 1.01 | |
| Smoking | 0.561449 | | 0.410734 | | 1.37 | | 0.172 | | 1.75 | | 0.78 | | 3.92 | |

**Coef** = Regression coefficient; **SE Coef** = Standard error of the coefficient; **Z** = Test statistic (Coef ÷ SE); **P** = P-value; OR = **Odds Ratio; 95% CI Lower / Upper** = Confidence interval for the OR.
