# Supplementary material for: Impact of GST and TP53 gene polymorphisms on susceptibility to end stage renal disease: A case-control analysis
Source: PLoS One. 2026 Oct 5;21(10):e0359809. doi: 10.1371/journal.pone.0359809 (PMC13637908; doi:10.1371/journal.pone.0359809)
Supplement: Supplementary file 7 [file pone.0359809.s007.doc]

**Supplementary Figure 1.**

Agarose gel electrophoresis based genotyping results.

**Legend**

A. Multiplex PCR based amplification of *GSTT1* and M1 gene. All lanes presenting 312 bp band specific for *CyP1A1* gene (control). Lanes presenting 480 band specific for *GSTT1* gene. Lanes presenting 218 bp band specific for *GSTM1* gene. DNA ladder of 100 bp and in some gels 50 bp was used for size comparison.

B. T-ARMS PCR based genotyping at rs1695 in *GSTP1*. Lanes presenting 290 bp band specific for mutant genotype (GG) and lanes presenting 233 bp band specific for wild genotype (AA). While lanes showing both 233 and 290 bp specific for heterozygous (AG) genotype. DNA ladder of 100 bp and in some gels 50 bp was used for size comparison.

C. T-ARMS PCR based genotyping at rs1042522 in *TP53*. DNA ladder of 100 bp and in some gels 50 bp was used for size comparison. Lanes presenting 200 bp band specific for wild genotype (GG), lanes presenting 247 bp specific for mutant genotype (CC) while lanes showing both 200 and 247 bp specific for heterozygous (CG) genotype.

**GSTT1/GSTM1 gel pictures**

**rs1695 in *GSTP1* gel pictures**

**rs1042522 in *TP53* gel pictures**
